# Abnormality of the Corpus Callosum in Coalmine Gas Explosion-Related Posttraumatic Stress Disorder

**DOI:** 10.1371/journal.pone.0121095

**Published:** 2015-03-23

**Authors:** Yang Zhang, Huabing Li, Xu Lang, Chuanjun Zhuo, Wen Qin, Quan Zhang

**Affiliations:** 1 Department of Radiology and Tianjin Key Laboratory of Functional Imaging, Tianjin Medical University General Hospital, Tianjin, 300052, China; 2 Department of Radiology of Jinmei Group General Hospital, Jincheng, Shanxi, 048006, China; 3 Department of Psychiatry, Anning Hospital of Tianjin City, Dongli District, Tianjin, 300300, China; University of Medicine & Dentistry of NJ—New Jersey Medical School, UNITED STATES

## Abstract

Abnormal corpus callosum (CC) has been reported in childhood trauma-related posttraumatic stress disorder (PTSD); however, the nature of white matter (WM) integrity alterations in the CC of young adult-onset PTSD patients is unknown. In this study, 14 victims of a coal mine gas explosion with PTSD and 23 matched coal miners without experiencing the coal mine explosion were enrolled. The differences in fractional anisotropy (FA) within 7 sub-regions of the CC were compared between the two groups. Compared to the controls, PTSD coal miners exhibited significantly reduced FA values in the anterior sub-regions of the CC (*P* < 0.05, Bonferroni-corrected), which mainly interconnect the bilateral frontal cortices. Our findings indicated that the anterior part of the CC was more severely impaired than the posterior part in young adult-onset PTSD, which suggested the patterns of CC impairment may depend on the developmental stage of the structure when the PTSD occurs.

## Introduction

Posttraumatic stress disorder (PTSD) is a chronic, debilitating psychiatric disorder that is characterized by symptoms of re-experiencing, hyperarousal, and avoidance. PTSD typically occurs in individuals who have experienced fear, helplessness, or horror following threat of injury or death [1]. Many neuroimaging studies have found differences in brain structures [2] and functions [3] between individuals with and without PTSD. Most previous research has focused on functional alterations of the amygdala, medial prefrontal cortex (PFC) and hippocampus [4–7]. However, little attention has been paid to white matter (WM) integrity changes in patients with PTSD [8].

The most consistent finding in terms of WM structures has been the association between childhood trauma-induced PTSD and decreased volume or fractional anisotropy (FA) values in the corpus callosum (CC). Diffusion tensor imaging (DTI) has revealed reduced FA values in the genu and body of the CC in childhood trauma-induced PTSD in adolescents [9] and in the midbody [10] and posterior regions [11] of the CC in PTSD children. Volumetric analysis also demonstrated a volume reduction in the total CC and its sub-regions in children with PTSD [12–14] and adults with childhood trauma-induced PTSD (Villarreal et al., 2004). However, the affected sub-regions of the CC are different across studies.

Studies on WM in adult-onset PTSD have revealed inconsistent results. Adult-onset PTSD patients had reduced FA in left anterior cingulum [15,16], right posterior cingulum [17], PFC and posterior angular gyrus [18], as well as increased FA in superior frontal gyrus [19] and left anterior cingulum [20]. Recently, reduced CC volume has also been reported in adults with PTSD [21]. However, there have been no studies that specifically examine WM integrity of the CC in young adult-onset PTSD.

It has been demonstrated that there is plasticity of the WM in adulthood, including myelination of previously unmyelinated axons and remodeling of existing myelin sheaths [22]. Thus, the WM of young adults is also at risk of damage when these individuals are exposed to the pathological condition. Neurodevelopmental arrest [23], neurodegenerative changes [24] or synaptic plasticity [25] have already been reported in adults who have experienced long-term pathological exposure. The maturation level of the CC in young adults is higher than that in children, and the vulnerability of the CC to traumatic events in young adults may likewise be different. Therefore, it is plausible to hypothesize that young adult-onset PTSD would also affect the WM integrity of CC and to some extent cause a different pattern of alterations compared with what is observed in child PTSD.

To test this hypothesis, a group of male coalminers with gas explosion-induced PTSD and matched coalminers without experiencing the coal mine explosion were recruited, and the intergroup differences in FA of the whole CC and its sub-regions were compared in this study. The coalmine gas explosion-related PTSD patients experienced the same trauma and had the same working environment and socioeconomic background, aiding in the identification of the PTSD related brain structural changes.

## Methods

### Subjects

This study included 24 coalminers who suffered from the coalmine gas explosion that occurred in February 2006 in Shanxi province, China and were diagnosed as PTSD within 6 months after the accident. These subjects were reviewed by two experienced psychiatrists 82 months later. Twenty-two of the miners were diagnosed with current and lifetime PTSD according to the Diagnostic and Statistical Manual of Mental Disorders (DSM-IV) criteria [26] and received MRI scanning in December 2012. All of the PTSD coalminers were carefully screened by clinical doctors to exclude participants with clinical signs and symptoms related to traumatic brain injury (TBI), and 8 of 22 PTSD coalminers were excluded because of visible brain lesions on conventional MR images (WM lesions, tumor and cyst). Twenty-five male coalminers without disaster experience were included as controls. Two of the twenty-five controls were excluded because of morphological variation in the CC (extremely narrow isthmus of the CC). In total, 14 PTSD patients and 23 controls were analyzed in the study. All the subjects denied trauma history prior to the coalmine gas explosion, and none of the subjects had substance abuse in the last 3 months. The Clinician Administered PTSD Scale (CAPS) [27] was administered to assess the symptom severity of each patient. The anxiety and depression symptoms of each subject were assessed using the Hamilton Rating Scales for Anxiety (HAMA) [28] and Depression (HAMD) [29], respectively.

The study was approved by the Ethical Committee of Tianjin Medical University General Hospital. We fully explained the aim and methods of the present study to all the subjects and their guardians. After acquiring the authority from the guardians, forensic psychiatrist evaluated and assured that all the subjects had civil capability and were willing to participate the study. Based on the aforementioned procedure, the informed consent was signed by both subjects and their guardians.

### MRI acquisition

MR images were obtained on a 3.0-Tesla scanner (Magnetom Verio, Siemens, Erlangen, Germany). DTI was performed using single-shot spin-echo echo planar imaging sequence, with the following parameters: repetition time = 6000 ms, echo time = 90 ms, flip angle = 90°, matrix = 128 × 128, field of view = 25.6 cm × 22.4 cm, number of excitation = 2, slice thickness = 3 mm, 44 slices with no gap, voxel dimension = 2 mm × 2 mm × 3 mm. The diffusion-sensitizing gradients were applied along 30 non-collinear directions, with a b value of 1000 s/mm2, and one volume was also acquired without diffusion weighting (b = 0).

### Data processing

The DTI data were preprocessed using FSL 4.1 (http://www.fmrib.ox.ac.uk/fsl). First, all of the diffusion volumes were aligned to the b0 images with an affine transformation to minimize image distortion by eddy currents and to reduce inter-volume head motion. Then, the brain tissues were extracted using FSL’s Brain Extraction Tool (BET) (http://www.fmrib.ox.ac.uk/fsl/bet2/index.html). Lastly, DTI data were interpolated into 2 mm×2 mm×2 mm voxels.

**Fig 1:**
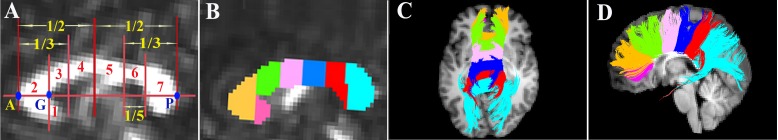
Sub-regions of the corpus callosum (CC). **A**, Mid-sagittal MR image of the CC showing the method (Witelson 1989) for defining 7 sub-regions. A and P indicate the anteriormost and posteriormost points of the CC. G, the anteriormost point on the inner convexity of the anterior callosum. A-P was used as the primary axis, lines perpendicular to which subdivide the CC into 7 sub-regions. **B**, Seven sub-regions were drawn as 7 seeds for fiber tracking. **C** and **D**, Fibers crossing through each seed on axial and sagittal anatomical images.

After the preprocessing steps, a Gaussian tensor model was fitted to each voxel using Diffusion Toolkit (www.trackvis.org), and a FA map was generated. The tractography was performed using the Fiber Assignment by Continuous Tracking (FACT) algorithm with an angular threshold of 40° and a FA threshold of 0.18. The CC sub-regions were defined on the mid-sagittal section of the FA maps using TrackVis software (www.trackvis.org). Two trained raters who were blind to subjects' identity segmented each CC into 7 sub-regions with a semi-automatic program **(Fig. 1)**. The details of this method were described by Witelson [30]. WM fibers through the total CC area and 7 sub-regions were tracked separately. The voxels of the 8 WM fibers were extracted as tracts-of-interest (TOI), and the average FA value of each TOI for each subject was extracted. In addition, the average FA values of the 6 mm-thick midsagittal section (ROI) of the 8 fibers were also extracted to verify the results of the total fibers. The intraclass correlations (ICC) of inter-rater measures ranged from 0.85 to 0.99, implying good inter-rater reliability **(Table 1)**. The average values of the two raters' manual measurements were used for further statistical analyses.

**Table 1:** Intra-rater reliability for FA values in corpus callosum.

Region	FA of the TOI		FA of the ROI	
	*ICC*	*P*	*ICC*	*P*
Whole CC	0.96	<0.001	0.99	<0.001
Sub-region 1	0.94	<0.001	0.95	<0.001
Sub-region 2	0.98	<0.001	0.98	<0.001
Sub-region 3	0.95	<0.001	0.99	<0.001
Sub-region 4	0.85	<0.001	0.99	<0.001
Sub-region 5	0.96	<0.001	0.98	<0.001
Sub-region 6	0.93	<0.001	0.98	<0.001
Sub-region 7	0.98	<0.001	0.99	<0.001

CC, corpus callosum; FA, Fractional Anisotropy; ICC, Intra-class coefficients; ROI, region of interested in midssagital setion; TOI, tracts of interest

Sub-regions 1–7 represent rostrum, genu, rostral body, anterior mid-body, posterior mid-body, isthmus, and splenium of the CC, respectively

**Fig 2:**
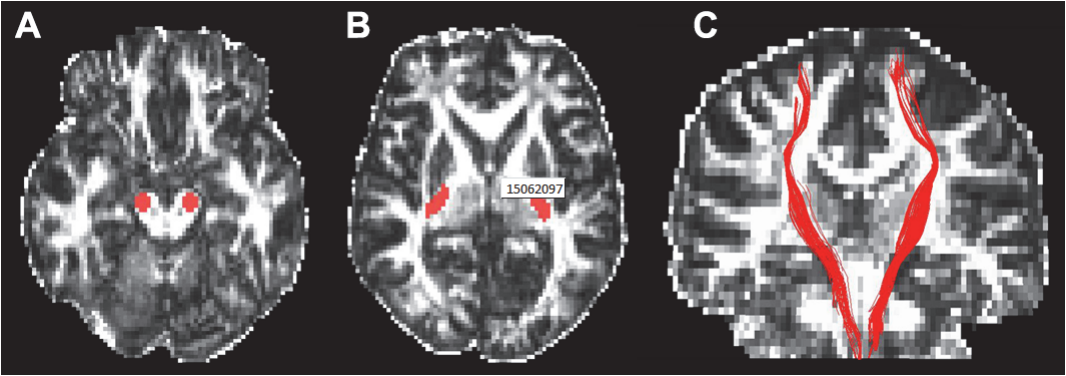
Locations of the seed regions of interest for tracing the corticospinal tract (A and B) and demonstration of the corticospinal tract (C) overlaied on the fractional anisotropy image.

To exclude the possibility that abnormal WM integrity in the CC was general damage resulting from the trauma itself or gas poisoning, the WM integrity of the bilateral corticospinal tracts (CST) were also evaluated as an additional control. These tracts were chosen because the CST is not a critical target of PTSD; however, it has been found to be impaired in carbon monoxide poisoning [31] or traumatic brain injury [32]. The tracing method for the CST was the same as that used in the CC. The seed regions were placed in the bilateral cerebral peduncles and posterior limbs of the internal capsules (**Fig. 2**). The ICCs of inter-rater measures were 0.95 and 0.90 for the left and the right CST, respectively. The average values of the two raters' manual measurements were used for further statistical analyses.

### Statistical analyses

All of the statistical analyses were performed using SPSS 21.0 (SPSS, Inc., Chicago.IL). Intergroup differences in demographic and clinical variables were examined using two-sample *t*-test. The inter-group differences in FA values of the 8 TOIs and 8 ROIs of the CC and bilateral CST were compared using two-sample *t*-tests with Bonferroni correction for multiple comparisons (*P* < 0.05). Pearson’s correlation was performed between the FA values and the CAPS in the PTSD group. The correlation analyses were also performed between the FA values and HAMD and HAMA scores in both groups. The significance level was set at *P* < 0.05.

## Result

There were no significant differences in age, educational levels or HAMA between the two groups (*P* > 0.05), but the HAMD score was higher in PTSD patients than in controls (*P* < 0.001). The demographic and clinical data are shown in **Table 2**.

**Table 2:** Socio-demographic characteristics of subjects (Mean±SD).

	PTSD (*n* = 14)	Control (*n* = 23)	*t*	*P*
Age (years)	33.1 ± 5.4	36.2 ± 5.3	−1.732	0.920
Education (years)	10.1 ± 1.5	9.8 ± 1.3	0.592	0.559
HAMA	14.9 ± 5.2	15.9 ± 1.9	−0.736	0.473
HAMD	14.5 ± 3.9	2.3 ± 2.3	12.005	**<0.001**
CAPS lifetime	87.6 ± 14.1			
CAPS current	50.4 ± 18.7			

CAPS, Clinician-Administered PTSD Scale; HAMA, Hamilton Anxiety Rating Scale; HDMD, Hamilton Depression Rating Scale; PTSD, posttraumatic stress disorder.

Compared with controls, PTSD patients exhibited a decreased FA value in the whole CC (*P* < 0.05). Following Bonferroni correction, PTSD patients exhibited reduced FA values in TOIs of the anterior 4 sub-regions, with effect sizes ranging from medium to strong (0.73–0.93) (Table 3), according to Cohen (1992) [33]. In addition, the differences in the FA values of the ROIs also survived Bonferroni correction in sub-regions 1 and 3, with effect sizes of 0.77 and 0.83, respectively (Table 4). There were no significant intergroup differences in the FA values of the left (PTSD: 0.58 ± 0.02; Controls: 0.57 ± 0.01; *t* = 0.591, *P* = 0.559) or the right CST (PTSD: 0.57 ± 0.02; Controls: 0.58 ± 0.02; *t* = -0.941, *P* = 0.353).

**Table 3:** Fractional anisotropy values of the tract of interest (TOI) in corpus callosum (Mean±SD).

Regions	PTSD (n = 14)	Control (n = 23)	*t*	*P*	Effect size (*Cohen's d*)
Whole CC	0.47±0.017	0.48±0.015	2.641	**0.012**	0.76
Sub-region 1	0.45±0.025	0.48±0.029	3.056	0.006*	0.75
Sub-region 2	0.45±0.016	0.47±0.015	3.119	0.004*	0.81
Sub-region 3	0.45±0.022	0.48±0.023	3.378	0.002*	0.93
Sub-region 4	0.47±0.027	0.49±0.021	3.015	0.005*	0.73
Sub-region 5	0.46±0.022	0.47±0.021	1.177	0.240	0.32
Sub-region 6	0.46±0.025	0.48±0.027	2.073	0.050	0.63
Sub-region 7	0.50±0.021	0.50±0.017	0.910	0.369	0.28

* Significance after Bonferroni-correction (*P* < 0.05/7); Sub-regions 1–7 represent rostrum, genu, rostral body, anterior mid-body, posterior mid-body, isthmus, and splenium of the corpus callosum, respectively.

**Table 4:** Mid-sagittal fractional anisotropy values of the region of interest (ROI) in corpus callosum (Mean±SD).

Regions	PTSD (*n* = 14)	Control (*n* = 23)	*t*	*p*	Effect size (*Cohen's d)*
Whole CC	0.49±0.033	0.52±0.035	3.361	0.002	0.80
Sub-region 1	0.47±0.041	0.52±0.065	3.059	0.004*	0.77
Sub-region 2	0.51±0.040	0.55±0.056	2.538	0.025	0.58
Sub-region 3	0.46±0.043	0.51±0.046	3.437	0.002*	0.83
Sub-region 4	0.47±0.043	0.51±0.065	2.475	0.018	0.62
Sub-region 5	0.45±0.054	0.49±0.051	2.114	0.042	0.50
Sub-region 6	0.48±0.076	0.54±0.071	2.352	0.024	0.56
Sub-region 7	0.57±0.068	0.58±0.065	0.616	0.542	0.15

* Significance after Bonferroni-correction (*P* < 0.05/7).

Sub-regions 1–7 represent rostrum, genu, rostral body, anterior mid-body, posterior mid-body, isthmus, and splenium of the corpus callosum, respectively.

There were no significant correlations between the FA values of any of the TOIs or ROIs and the CAPS scores in the PTSD group. Likewise, no significant correlations were observed between the FA values and the HAMD or HAMA scores in either group.

## Discussion

In the present study, we used DTI to investigate the abnormalities of the CC in young adult-onset PTSD. PTSD patients exhibited significantly decreased FA values in the anterior part of the CC, which connects the bilateral prefrontal cortices.

The corpus callosum is the largest WM fiber tract that connects homologous cortical regions of the bilateral hemispheres [34]. There is a topological correspondence between the CC fibers and their corresponding cortical regions from which these fibers originate [30]. Thus, we analyzed WM integrity of the whole CC to reflect the gross involvement of this structure in young adult-onset PTSD. We also investigated WM integrity of CC sub-regions to determine which sub-regions were more affected in the young adult-onset PTSD.

In this study, we analyzed WM integrity of the CC using TOI and ROI methods. The FA value of a TOI is not only affected by the tract of interest but also by its neighboring or crossing fibers. The ROIs of the CC are located in midsagittal regions, where the arrangement of the CC is highly consistent, resulting in a more accurate measurement of the FA value.

In this study, non-PTSD coalminers also had high HAMA scores that were similar to the PTSD coalminers, which might be caused by chronic stress caused by the coal mine environment. We chose non-PTSD coalminers as controls to exclude any factors that may coexist in both the controls and PTSD patients that might interfere with the results. These factors included gender, age, education, working environment, general mental status, labor intensity and living community, etc. The HAMD scores were higher in PTSD coalminers than in non-PTSD coalminers in present study. There is a high level of comorbidity between PTSD and depression [35], and attempts to control for the comorbidity may risk removing effects that are actually those of interest [36]. Therefore, the effect of comorbid depression was not controlled in our study in order to reflect the generalizability of findings in the young adult-onset PTSD patients.

Decreased FA was found in the anterior part of the CC in young adult-onset PTSD coalminers in this study. The anterior part of the CC constitutes an inter-hemispheric connection between the frontal cortices and plays an important role in inter-hemispheric transfer of signals [37].

The decreased FA in the CC may result from high levels of corticosteroids in PTSD patients [38]. This can be hypothesized because the CC is susceptible to the impact of exposure to high levels of stress hormones, which suppress the glial cell division that are critical for myelination [39]. The reduced integrity of anterior part of CC may be associated with the structural and functional alteration of the PFC that have been found in previous PTSD studies [2,3,40,41]. The impairment of WM and gray matter may influence each other bi-directionally in the pathological state of PTSD. On the one hand, CC abnormalities may affect the function and structure of PFC. For example, it has been reported that reduced size of the CC is associated with reduced communication between the cortices of the two hemispheres [42], and delayed myelination of the CC may also affect the cortical development of the hemispheres [39]. On the other hand, decreased integrity of CC may result from the axonal degeneration secondary to the PFC atrophy that has been reported in previous PTSD studies [43]. Although the relationship between the WM integrity of the anterior part of CC and the PFC has yet to be clearly elucidated, it can be postulated that abnormal WM integrity in the anterior part of CC may lead to reduced structural connections between the bilateral prefrontal cortices, which may be associated with the pathophysiological changes of the PTSD.

There was no significant correlation between the FA values of the CC and PTSD symptoms in this study. A possible explanation for this result may be that the decreased integrity of the anterior CC mainly causes a reduced connection between the bilateral PFCs and has no direct relation to the symptom severity of PTSD. In addition, we could not exclude the possibility that decreased FA values in the anterior CC were only a trait marker, not a state marker, of PTSD.

In contrast to the severe impairment of the WM integrity of the anterior part of the CC, there was no significant intergroup difference in WM integrity in the posterior part of the CC in young adult-onset PTSD after correction for multiple comparisons. However, a trend towards reduced FA in posterior part of the CC was detected with an uncorrected threshold. This finding is inconsistent with previous studies showing significantly decreased FA values in these regions in patients with childhood-onset PTSD [10,11]. One possible explanation for the discrepancy between the young adult-onset and child PTSD is the different maturity levels of the CC between these groups.

The CC has been observed to develop into the mid-20s [44], with a posterior to anterior myelination order [23]. This pattern suggests that different sub-regions of CC have different time windows of vulnerability to negative experiences [12,45]. In childhood, the CC is not mature and is more vulnerable to negative experiences, such as stress. This mechanism may underlie the reduced CC integrity in childhood-onset PTSD. However, the PTSD coalminers enrolled in this study were exposed to the disaster at young adulthood, when most of the brain tissues are well developed, except for the highest-order/latest-maturing brain regions (bilateral PFC) [39,46] and the WM tracts interconnecting them (anterior part of CC) [23,44]. The anterior part of the CC is still myelinating or remodeling in young adulthood and is more vulnerable to negative experience than posterior regions; this fact may explain our observation of more severely damaged WM in the anterior part of the CC than in the posterior part.

The posterior CC exhibited only a trend towards reduced FA in the PTSD group in this study. The non-significant intergroup difference in FA in the posterior parts of the CC may partially be caused by the relatively small sample of the recruited PTSD patients; this trend may reach significance with a larger sample. Thus, our results did not exclude the possibility that posterior CC was also involved in young adult-onset PTSD. Alternatively, our result strongly suggests that the anterior CC had a more severe impairment than the posterior CC when the PTSD occurred at a relatively late stage (young adulthood).

We were not able to study a control group that had been exposed to the same gas explosion but had not developed into PTSD, which would have allowed us to exclude the potential toxic and physical effects of the event. However, there were no significant differences in the FA of either the left or right CST between the PTSD and healthy controls, implying that the changes in WM integrity in the CC may reflect the specific damage by PTSD rather than general damage by gas poisoning or trauma. Although we did not correct the partial volume effect (PVE) during calculating the FA values in this study, which may affect the accuracy of the FA values in the voxels, especially the outer voxels within ROIs [47], the PVE is relatively small in the CC because of its big volume, and our results could still reflect the association between decreased FA values in CC and the PTSD. Another limitation of this study was that negative alterations in cognition and mood, which are included in the recent DSM-V, were not evaluated in the PTSD group because the data collection of this study was complete in 2012, when the DSM-V had not been published; therefore, our diagnostic criteria of PTSD were based on DSM-IV.

## Conclusions

To our knowledge, this is the first study using DTI method to investigate the integrity of CC in young adult-onset PTSD. We found that adult-onset PTSD patients exhibited more severe impairment of WM integrity in the anterior CC relative to that of the posterior CC, which is different from the alteration pattern in child PTSD reported in the literatures. These findings suggest that the patterns of CC impairment may depend on the developmental stage of the affected structure when the PTSD occurs.
